# Disclosure of same-sex practices and experiences of healthcare stigma among cisgender men who have sex with men in five sub-Saharan African countries

**DOI:** 10.1186/s12889-021-12151-3

**Published:** 2021-12-03

**Authors:** John Mark Wiginton, Sarah M. Murray, Ohemaa Poku, Jura Augustinavicius, Kevon-Mark Phillip Jackman, Jeremy Kane, Serge C. Billong, Daouda Diouf, Ibrahima Ba, Tampose Mothopeng, Iliassou Mfochive Njindam, Gnilane Turpin, Ubald Tamoufe, Bhekie Sithole, Maria Zlotorzynska, Travis H. Sanchez, Stefan D. Baral

**Affiliations:** 1 Department of Health, Behavior & Society, Johns Hopkins University Bloomberg School of Public Health, 624 N Broadway Street, Baltimore, MD 21205 USA; 2 Department of Mental Health, Johns Hopkins University Bloomberg School of Public Health, Baltimore, MD USA; 3 Department of Epidemiology, Columbia University Mailman School of Public Health, New York City, NY USA; 4 Department of Public Health, Faculty of Medicine and Biomedical Sciences, University of Yaoundé I, Yaoundé, Cameroon; Central Technical Group, National AIDS Control Committee, Yaoundé, Cameroon; 5 Enda Santé, Dakar, Senegal; 6 The People’s Matrix, Maseru, Lesotho; 7 Center for Public Health & Human Rights, Department of Epidemiology, Johns Hopkins University Bloomberg School of Public Health, Baltimore, MD USA; 8 Metabiota, Yaounde, Cameroon, Johns Hopkins Cameroon Program, Yaounde, Cameroon; 9 Mbabane, FHI 360, Mbabane, eSwatini; 10 Department of Epidemiology, Emory University Rollins School of Public Health, Atlanta, GA USA

**Keywords:** Disclosure, Healthcare stigma, Men who have sex with men, Sub-Saharan Africa

## Abstract

**Background:**

For men who have sex with men (MSM) across sub-Saharan Africa (SSA), disclosure of same-sex practices to family and healthcare workers (HCWs) can facilitate access to HIV prevention services and support, but can also lead to experiences of stigma.

**Methods:**

We performed mixed-effects regressions on pooled data from MSM in Cameroon, Senegal, Côte d’Ivoire, Lesotho, and eSwatini to assess associations between disclosure and sexual behavior stigma in healthcare contexts; we used logistic regressions to analyze country-specific data.

**Results:**

Compared to participants who had not disclosed to either family or HCWs, those who had disclosed only to family were more likely to have been gossiped about by HCWs (aOR = 1.70, CI = 1.18, 2.45); the association between having disclosed to family and having felt mistreated in a health center approached, but did not achieve, statistical significance (aOR = 1.56, CI = 0.94, 2.59). Those who had disclosed only to HCWs were more likely to have feared to seek health services (aOR = 1.60, CI = 1.14, 2.25), avoided health services (aOR = 1.74, CI = 1.22, 2.50), and felt mistreated in a health center (aOR = 2.62, CI = 1.43, 4.81). Those who had disclosed to both were more likely to have feared to seek health services (aOR = 1.71, CI = 1.16, 2.52), avoided health services (aOR = 1.59, CI = 1.04, 2.42), been gossiped about by HCWs (aOR = 3.78, CI = 2.38, 5.99), and felt mistreated in a health center (aOR = 3.39, CI = 1.86, 6.20). Country-specific analyses suggested that data from Cameroon drove several of these associations.

**Conclusions:**

Research to determine the factors driving disclosure’s differential effect on healthcare stigma across contexts is needed. Ultimately, supportive environments enabling safe disclosure is critical to understanding HIV-acquisition risks and informing differentiated HIV-prevention, treatment, and testing services for MSM across SSA.

**Supplementary Information:**

The online version contains supplementary material available at 10.1186/s12889-021-12151-3.

## Background

Globally, men who have sex with men (MSM) continue to bear a disproportionate burden of HIV [1]. This burden is in part driven and exacerbated by intersecting social stigmas related to both same-sex practices and HIV [2–4]. These stigmas are especially severe in settings in the 36 countries across sub-Saharan Africa (SSA) that criminalize same-sex practices [4–7]. Sexual behavior stigma undermines HIV prevention efforts, including HIV testing, uptake of and adherence to HIV prevention and treatment medication, and engagement in other sexual health services [8, 9].

One mechanism through which sexual behavior stigma impedes HIV prevention is enacted healthcare stigma, which involves healthcare workers’ (HCWs) overt discrimination (e.g., denial of services) and mistreatment (e.g., verbal harassment, gossip) of MSM due to engagement in same-sex practices [10, 11]. Experiences of enacted healthcare stigma can disrupt HIV service utilization and dissuade MSM from future help-seeking [12, 13]. Sexual behavior stigma may further constrain healthcare access through perceived or anticipated healthcare stigma, which includes the perception, expectation, or fear of current or future mistreatment by HCWs due to one’s engagement in same-sex practices [14–16]. Such stigma can lead to missed opportunities for preventive screening and other services, thereby increasing risk for HIV and sexually transmitted infections [17–19].

Experiences of sexual behavior stigma among MSM in healthcare contexts have often been linked to disclosure of engagement in same-sex practices to HCWs [7, 9,20–23]. The relationship between healthcare stigma and disclosure of same-sex practices to family has been less researched, perhaps due to the more indirect, complex pathways between the two. Scholarship has linked family disclosure to difficulties accessing health services for MSM in SSA [24], possibly reflecting the loss of social and financial support from family following disclosure, which may explain why disclosure by MSM to family has also been linked to economic independence [23, 25]. Alternatively, the link between family disclosure and difficulties accessing healthcare could be due to perceived or anticipated healthcare stigma stemming from family disclosure experiences. That is, negative reactions from family following disclosure may prime MSM to anticipate or perceive similar reactions from HCWs, avoiding disclosure to HCWs or avoiding healthcare encounters altogether [23, 26]. Positive reactions from family following disclosure could also lead to healthcare stigma. MSM who feel validated and supported by their family may be less concerned with concealing their sexuality in other contexts, proudly disclosing their same-sex practices to HCWs or displaying gender nonconforming mannerisms that may be perceived as homosexual, incurring enacted stigma as a result [23, 27]. While non-disclosure could perhaps be protective against some of the impacts of sexual behavior stigma [28–30], it could also preclude MSM from reaping disclosure’s benefits. Research has repeatedly established links between disclosure of same-sex practices by MSM to HCWs and receipt or utilization of appropriate HIV care and other sexual health services [31–33]. Similarly, disclosure to family may yield acceptance and affirmative social support, facilitate disclosure to HCWs, promote engagement in sexual health services, and support the adoption of positive health behaviors [30, 34–39].

Given the complex roles of disclosure across healthcare and family contexts, there remains a need to better understand how and the extent to which different disclosures are related to stigma within healthcare settings. We therefore assessed patterns of disclosure of same-sex practices to family and HCWs by cisgender MSM in five sub-Saharan African countries (Cameroon, Senegal, Côte d'Ivoire, Lesotho, eSwatini) and examined how such patterns may be differentially linked to multiple forms of healthcare stigma. 

## Methods

This study involved secondary analysis of cross-sectional data collected from cisgender MSM between 2013 and 2016 using respondent-driven sampling (RDS) (Cameroon, Senegal, eSwatini, Côte d’Ivoire) or snowball sampling (Lesotho). In the countries where RDS was used, 3–16 seeds selected by/from community-based organizations recruited participants across 12–20 waves of recruitment [4, 40–45]. Given that the lived experience of being transgender or nonbinary is distinct from that of being cisgender, we excluded data from gender minorities in this study, which require separate analyses focused on intersectional stigmas [44, 46]. Cisgender men age ≥ 18 years reporting past-year anal sex with a man completed a face-to-face survey assessing sexual behavior stigma, sexuality disclosure, HIV diagnosis and treatment, and sociodemographic and other characteristics. Participants received US$2–$6 for their time and travel costs, and for each eligible RDS recruit. Studies were conducted in compliance with local ethical standards and received the approval of local ethics committees and Johns Hopkins University institutional review board. This secondary analysis of deidentified data received exemption from Johns Hopkins University institutional review board. Detailed data collection methods have been described elsewhere [4, 40–45].

### Measures

Outcome variables were four yes/no indicators of sexual behavior stigma related to the healthcare system: (1)“Have you ever felt afraid to go to healthcare services because you worry someone may learn you have sex with men?” (2) “Have you ever avoided going to healthcare services because you worry someone may learn you have sex with men?” (3) Have you ever heard healthcare providers gossiping about you (talking about you) because you have sex with men?” (4) “Have you ever felt that you were not treated well in a health center because someone knew that you have sex with men?” [4, 33, 40, 47, 48]. Items 1–2 assessed anticipated healthcare stigma, and items 3–4 assessed enacted and perceived healthcare stigma. The exposure variable, disclosure of same-sex practices, was assessed using two yes/no items that asked if participants had ever told a (1) HCW or (2) family member about their engagement in same-sex practices.

Control variables included sociodemographic and other characteristics. Age was measured in years. Education was dichotomized into having completed secondary school or beyond versus not having finished secondary school. A dummy variable was created to indicate country of residence, and income quintiles were created based on within-country income comparisons. Self-reported HIV status was measured with three response options: positive, negative, and unknown. Depression, which was included due to its links to healthcare stigma and sexual behavior disclosure among MSM in other sub-Saharan African contexts [12, 19, 28, 30], was measured with the Patient Health Questionnaire-9 [49, 50]. This questionnaire assessed the frequency of nine depressive symptoms within the previous two weeks. Response options included “not at all” (0), “several days” (1), “more than half the days” (2), and “nearly every day” (3). Items were summed, with a score of ≥5 indicative of at least mild depression [51].

### Analysis

Data from all countries were pooled and analyzed as crude data; RDS-adjusted weighting was not applied across countries as individuals did not represent a single network, violating a key assumption of RDS [52]. Frequency of endorsement and missingness for each stigma and disclosure item was assessed [15]. Descriptive statistics were calculated for sociodemographic and other variables, and chi-square tests (for categorical variables) and the Wilcoxon rank-sum test (for age) were used to compare differences in these variables by endorsement of each healthcare stigma item. Mixed-effects logistic regression models with a random effect for country were constructed to examine associations between healthcare stigma and disclosure. Initially, associations between each dichotomous disclosure variable and each healthcare stigma outcome variable were examined, with product terms (disclosure to a HCW*disclosure to a family member) being incorporated to assess multiplicative interaction effects between both types of disclosure and healthcare stigma. However, as none of the interaction terms were significant in the pooled analysis and were excluded, we instead combined responses to create a four-level categorical variable: (1) have not disclosed to a HCW or family member (reference); (2) have disclosed to a family member only; (3) have disclosed to a HCW only; (4) have disclosed to both a family member and HCW.

In building the mixed-effect regression models, we first created empty models to assess the presence of significant clustering of each healthcare stigma outcome within countries. Next, the categorical disclosure variable was added to each empty model to examine bivariate associations with each healthcare stigma variable. Multivariable models were subsequently constructed, with the inclusion of control variables. In addition, separate logistic regression analyses were performed on selected country-specific data. Coefficients were exponentiated to generate odds ratios, and Wald tests, with significance set at α = 0.05, and 95% confidence intervals were calculated and examined. Model results represent valid sample estimates but may not represent population-level estimates given the lack of RDS-adjustment. Procedures were performed in Stata Version 15 [53].

## Results

The total cross-country datasets included data from 4405 participants, 3284 of whom reported being a cisgender man and eligible for inclusion in this analysis; 244 participants were missing data on key variables and were excluded, resulting in a complete-case analysis sample size of *N* = 3040. Most participants were from Côte d’Ivoire or Cameroon, followed by Lesotho, eSwatini, and Senegal (Table 1). Median age was 24 years, over three-quarters reported high school or less education, and > 40% reported income in the lowest quintile. Roughly one in six participants had clinically significant depressive symptoms. Approximately 6% (172/3040) reported positive HIV status, and 25% (758/3040) reported unknown HIV status.

**Table 1:** Characteristics of MSM in SSA, 2014–2016 (N = 3040)

Country, n (%)	
Côte d’Ivoire	947 (31.2)
Cameroon	838 (27.6)
Lesotho	476 (15.7)
eSwatini	393 (12.9)
Senegal	386 (12.7)
Age in years	
Mean (SD)	24.5 (5.1)
Median (IQR)	24 (21–27)
Education, n (%)	
≤ High school	2386 (78.5)
> High school	654 (21.5)
Income, n (%)	
Quintile 1	1263 (41.5)
Quintile 2	460 (15.1)
Quintile 3	206 (6.8)
Quintile 4	627 (20.6)
Quintile 5	484 (15.9)
HIV status, n (%)	
Negative	2110 (69.4)
Positive	172 (5.7)
Unknown	758 (24.9)
Depression, n (%)	
Yes	490 (16.1)
No	2550 (83.9)
Disclosure of same-sex practices	
Not to a family member or a HCW, n (%)	2064 (67.9)
To a family member only, n (%)	566 (18.6)
To a HCW only, n (%)	243 (8.0)
To a family member and a HCW, n (%)	167 (5.5)

MSM, men who have sex with men; SSA, sub-Saharan Africa; SD, standard deviation; IQR, interquartile range; HIV, human immunodeficiency virus; HCW, healthcare worker

**Fig. 1:**
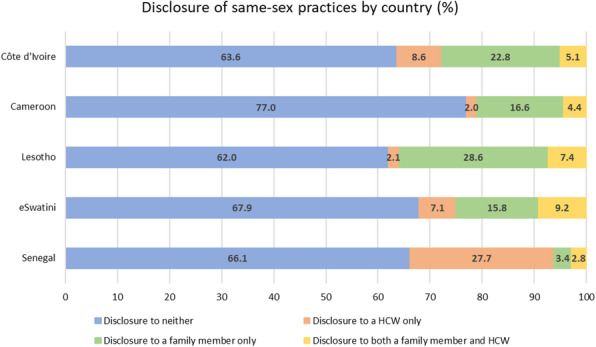
Disclosure of same-sex practices among MSM in SSA (by country), 2014–2016 (*N* = 3040). MSM, men who have sex with men; SSA, sub-Saharan Africa; HCW, healthcare worker

Overall, 67.9% (2064/3040) of participants had not disclosed their same-sex practices to a family member or HCW, 18.6% (566/3040) had disclosed to a family member only, 8.0% (243/3040) had disclosed to a HCW only, and 5.5% (167/3040) had disclosed to both. Disclosure to neither a family member nor a HCW was highest in Cameroon and lowest in Lesotho. Disclosure to a family member only was highest in Lesotho and lowest in Senegal. Disclosure to a HCW only was highest in Senegal and lowest in Cameroon. Disclosure to both was highest in eSwatini and lowest in Senegal (Fig. 1). Nearly one in five participants (539/3040) reported having felt afraid to seek health services, while one in seven (436/3040) reported having avoided health services due to worry over someone learning they have sex with men. Seven percent of participants (211/3040) reported hearing healthcare providers gossip about them because they have sex with men, and 4% (120/3040) reported having felt mistreated in a health center because they have sex with men (Table 2).

**Table 2:** Differences in sociodemographic and other characteristics by healthcare stigma among MSM in SSA, 2014–2016 (N = 3040)

	Afraid to seek health services		Avoided seeking health services		Gossiped about by healthcare providers		Felt mistreated in a health center	
	Yes (*n* = 539, 17.7%)	No (*n* = 2501, 82.3%)	Yes (*n* = 436, 14.3%)	No (*n* = 2604, 85.7%)	Yes (*n* = 211, 6.9%)	No (*n* = 2829, 93.1%)	Yes (*n* = 120, 3.9%)	No (*n* = 2920, 96.1%)
Country, n (%)^a^								
Côte d’Ivoire	196 (36.4)	751 (30.0)	127 (29.1)	820 (31.5)	81 (38.4)	866 (30.6)	24 (20.0)	923 (31.6)
Cameroon	82 (15.2)	756 (30.2)	79 (18.1)	759 (29.2)	40 (19.0)	798 (28.2)	25 (20.8)	813 (27.8)
Lesotho	51 (9.5)	425 (17.0)	35 (8.0)	441 (16.9)	15 (7.1)	461 (16.3)	16 (13.3)	460 (15.8)
eSwatini	144 (26.7)	249 (10.0)	138 (31.7)	255 (9.8)	57 (27.0)	336 (11.9)	45 (37.5)	348 (11.9)
Senegal	66 (12.2)	320 (12.8)	57 (13.1)	329 (12.6)	18 (8.5)	368 (13.0)	10 (8.3)	376 (12.9)
Chi-square *p*-value	< 0.001		< 0.001		< 0.001		< 0.001	
Age in years								
Mean (SD)	24.4 (4.9)	24.5 (5.3)	24.7 (4.7)	24.5 (5.3)	25.1 (5.3)	24.4 (5.2)	25.4 (5.5)	24.5 (5.2)
Median (IQR)	24 (21–27)	23 (21–27)	24 (21–27)	23 (21–27)	24 (21–28)	23 (21–27)	24 (22–28)	23 (21–27)
Wilcoxon p-value	0.067		< 0.010		< 0.010		< 0.05	
Education, n (%)								
≤ High school	386 (71.6)	2000 (80.0)	314 (72.0)	2072 (79.6)	151 (71.6)	2235 (79.0)	90 (75.0)	2296 (78.6)
> High school	153 (28.4)	501 (20.0)	122 (28.0)	532 (20.4)	60 (28.4)	594 (21.0)	30 (25.0)	624 (21.4)
Chi-square p-value	< 0.001		< 0.001		< 0.050		0.343	
Income, n (%)								
Quintile 1	230 (42.7)	1033 (41.3)	162 (37.2)	1101 (42.3)	81 (38.4)	1182 (41.8)	31 (25.8)	1232 (42.2)
Quintile 2	66 (12.2)	394 (15.8)	59 (13.5)	401 (15.4)	32 (15.2)	428 (15.1)	17 (14.2)	443 (15.2)
Quintile 3	50 (9.3)	156 (6.2)	44 (10.1)	162 (6.2)	19 (9.0)	187 (6.6)	20 (16.7)	186 (6.4)
Quintile 4	105 (19.5)	522 (20.9)	85 (19.5)	542 (20.8)	41 (19.4)	586 (20.7)	31 (25.8)	596 (20.4)
Quintile 5	88 (16.3)	396 (15.8)	86 (19.7)	398 (15.3)	38 (18.0)	446 (15.8)	21 (17.5)	463 (15.9)
Chi-square p-value	< 0.05		< 0.010		0.561		< 0.001	
HIV status, n (%)								
Negative	363 (67.4)	1747 (69.9)	294 (67.4)	1816 (69.7)	149 (70.6)	1961 (69.3)	78 (65.0)	2032 (69.6)
Positive	39 (7.2)	133 (5.3)	28 (6.4)	144 (5.5)	18 (8.5)	154 (5.4)	12 (10.0)	160 (5.5)
Unknown	137 (25.4)	621 (24.8)	114 (26.2)	644 (24.7)	44 (20.9)	714 (25.2)	30 (25.0)	728 (24.9)
Chi-square p-value	0.188		0.574		< 0.088		0.105	
Depression, n (%)								
Yes	161 (29.9)	329 (13.2)	149 (34.2)	341 (13.1)	71 (33.6)	419 (14.8)	51 (42.5)	439 (15.0)
No	378 (70.1)	2172 (86.8)	287 (65.8)	2263 (86.9)	140 (66.4)	2410 (85.2)	69 (57.5)	2481 (85.0)
Chi-square p-value	< 0.001		< 0.001		< 0.001		< 0.001	

MSM, men who have sex with men; SSA, sub-Saharan Africa; SD, standard deviation; IQR, interquartile range; HIV, human immunodeficiency virus

^a^Prevalence estimates for Cameroon, Senegal, eSwatini, and Côte d’Ivoire represent valid sample estimates but may not represent population-level estimates due to lack of adjustment for respondent-driven sampling

Compared to participants who had not disclosed to either a family member or HCW, those who had disclosed only to a family member were significantly more likely to have been gossiped about by healthcare providers; the association between having disclosed only to a family member and having felt mistreated in a health center approached, but did not achieve, statistical significance. Those who had disclosed only to a HCW were significantly more likely to have feared to seek health services, avoided health services, and felt mistreated in a health center. Those who had disclosed to both were significantly more likely to have feared to seek health services, avoided health services, been gossiped about by healthcare providers, and felt mistreated in a health center (Table 3).

**Table 3:** Associations between disclosure of same-sex practices and healthcare stigma among MSM in SSA (pooled), 2014–2016 (N = 3040)

	Afraid to seek health services			Avoided health services			Gossiped about by healthcare providers			Felt mistreated in a health center		
	Empty	OR (95% CI)	aOR (95% CI)	Empty	OR (95% CI)	aOR (95% CI)	Empty	OR (95% CI)	aOR (95% CI)	Empty	OR (95% CI)	aOR (95% CI)
Fixed effects												
Same-sex practices disclosure												
Not to a FM or a HCW (ref)	–	1.00	1.00	–	1.00	1.00	–	1.00	1.00	–	1.00	1.00
To a FM only	–	1.04 (0.80, 1.35)	1.04 (0.80, 1.35)	–	1.02 (0.77, 1.36)	1.04 (0.77, 1.39)	–	1.66** (1.16, 2.37)	1.70** (1.18, 2.45)	–	1.42 (0.86, 2.33)	1.56 ~ (0.94, 2.59)
To a HCW only	–	1.64** (1.18, 2.27)	1.60** (1.14, 2.25)	–	1.76** (1.24, 2.50)	1.74*** (1.22, 2.50)	–	1.45 (0.86, 2.45)	1.32 (0.77, 2.25)	–	2.79** (1.55, 5.03)	2.62** (1.43, 4.81)
To a FM and a HCW	–	1.93** (1.33, 2.79)	1.71** (1.16, 2.52)	–	1.77** (1.18, 2.65)	1.59* (1.04, 2.42)	–	4.14*** (2.67, 6.40)	3.78*** (2.38, 5.99)	–	4.44*** (1.95, 6.05)	3.39*** (1.86, 6.20)
Random effect												
Country-level variance (SE)	0.35*** (0.23)	0.34*** (0.22)	0.28*** (0.19)	0.42*** (0.28)	0.40*** (0.27)	0.31*** (0.21)	0.31*** (0.22)	0.30*** (0.21)	0.26*** (0.20)	0.35*** (0.25)	0.28*** (0.20)	0.19** (0.15)
Model characteristics												
Intra-class correlation	9.7%	9.3%	7.9%	11.4%	10.9%	8.6%	8.5%	8.3%	7.5%	9.7%	9.9%	5.3%

~*p* < 0.10; *p < 0.05; **p < 0.01; ***p < 0.001

MSM, men who have sex with men; SSA, sub-Saharan Africa; OR, odds ratio; aOR, adjusted odds ratio; CI, confidence interval; FM, family member; HCW, healthcare worker; SE, standard error

Controlling for age, education, income, HIV status, and depression

As residual variance due to country-level differences was significant and ranged from 5.3–8.6% across adjusted models (Table 3), we analyzed country-level data. However, exploratory analyses revealed small cell values of both disclosure and healthcare stigma experiences in most countries, with particularly small (and occasionally empty) cells in Lesotho and Senegal. We therefore restricted country-level regression analyses to Côte d’Ivoire and Cameroon, as each made up roughly 30% of the overall sample size. Analysis of Côte d’Ivoire data found some significant adjusted associations, which were positive and confined to perceived and enacted healthcare stigma (Table 4). Re-running the pooled country multilevel model without Côte d’Ivoire resulted in few changes compared to the pooled country multilevel model with Côte d’Ivoire included (see Additional Table 1).

**Table 4:** Associations between disclosure of same-sex practices and healthcare stigma among MSM in SSA (by country), 2014–2016 (N = 3040)

	Afraid to seek health services		Avoided seeking health services		Gossiped about by healthcare providers		Felt mistreated in a health center	
	OR (95% CI)	aOR (95% CI)	OR (95% CI)	aOR (95% CI)	OR (95% CI)	aOR (95% CI)	OR (95% CI)	aOR (95% CI)
Same-sex practices disclosure								
Not to a FM or a HCW (ref)	1.00	1.00	1.00	1.00	1.00	1.00	1.00	1.00
Côte d’Ivoire								
To a FM only	0.99 (0.67, 1.47)	0.95 (0.88, 2.64)	1.23 (0.78, 1.94)	1.19 (0.74, 1.89)	1.84* (1.09, 3.11)	1.79* (1.05, 3.04)	1.80 (0.69, 4.70)	1.75 (0.66, 4.63)
To a HCW only	1.73* (1.03, 2.90)	1.52 (0.88, 2.64)	1.96* (1.09, 3.52)	1.75 ~ (0.93, 3.30)	0.73 (0.25, 2.10)	0.58 (0.20, 1.74)	0.67 (0.09, 5.27)	0.31 (0.03, 3.15)
To a family and a HCW	1.37 (0.69, 2.71)	1.25 (0.61, 2.55)	1.26 (0.54, 2.91)	1.19 (0.49, 2.88)	3.68*** (2.26, 9.70)	4.16*** (1.93, 8.99)	6.25** (2.08, 18.80)	3.96* (1.17, 13.39)
Cameroon								
To a FM only	1.73 ~ (0.96, 3.12)	1.55 (0.85, 2.83)	1.18 (0.62, 2.23)	1.06 (0.55, 2.04)	1.87 (0.85, 4.14)	2.07 ~ (0.91, 4.71)	3.22* (1.29, 8.04)	3.53* (1.36, 9.19)
To a HCW only	6.78*** (2.40, 19.14)	5.59** (1.88, 16.57)	6.22** (2.21, 17.50)	4.84** (1.64, 14.28)	5.80** (1.56, 21.58)	3.62 ~ (0.89, 14.65)	7.03* (1.45, 34.22)	4.40 (0.74, 26.15)
To a FM and a HCW	5.26*** (2.45, 11.29)	4.37** (1.87, 10.19)	3.15** (1.37, 7.23)	2.25 ~ (0.90, 5.61)	4.23** (1.51, 11.84)	2.30 (0.73, 7.31)	4.65* (1.25, 17.27)	3.78 ~ (0.83, 17.30)

~p < 0.10; *p < 0.05; **p < 0.01; ***p < 0.001; significant, adjusted associations are also bolded

MSM, men who have sex with men; SSA, sub-Saharan Africa; OR, odds ratio; aOR, adjusted odds ratio; CI, confidence interval; FM, family member; HCW, healthcare worker

Controlling for age, education, income, HIV status, and depression

We repeated this process with Cameroon, with slightly more significant and several trending  adjusted associations emerging between disclosure and stigma in country-specific analyses. In the Cameroon data, compared to participants who had not disclosed to either a family member or HCW, those who had disclosed only to a family member were significantly more likely to have felt mistreated in a health center; the association between having disclosed only to a family member and having been gossiped about by healthcare providers approached, but did not achieve, statistical significance. Those who disclosed only to a HCW were significantly more likely to have feared to seek health services and avoided health services; the association between having disclosed only to a HCW and having been gossiped about by healthcare providers approached, but did not achieve, statistical significance. Those who had disclosed to both were significantly more likely to have feared to seek health services; the association between having disclosed to both and having avoided health services and having felt mistreated in a health center approached, but did not achieve, statistical significance (Table 4). Re-running the pooled country multilevel model without Cameroon revealed fewer significant associations compared to the pooled model that included data from Cameroon; these associations were positive and somewhat similar to those observed in the Cote D’Ivoire-only analysis (see Additional Table 2).

## Discussion

We assessed patterns of disclosure of same-sex practices and examined their associations with four experiences of healthcare stigma among cisgender MSM in Côte d’Ivoire, Cameroon, Lesotho, eSwatini, and Senegal. Notably, a large majority of participants in each country had not disclosed their engagement in same-sex practices to either a family member or HCW. In pooled-country analyses, we detected several positive associations between disclosure to a family member, HCW, or both and each form of healthcare stigma. Country-specific analyses revealed that several of the associations observed between stigma and disclosure were driven by data from Cameroon, where non-disclosure was highest.

Among disclosure patterns, disclosure only to a family member was by far the most common in all countries except Senegal. This finding, coupled with the otherwise non-disclosing trend, showcases the rarity of disclosing one’s same-sex practices to HCWs. Such non-disclosure to HCWs necessarily constrains one’s access to and utilization of HIV-preventive and other sexual health services [8, 9]. Interventions facilitating safe, private disclosure that also train healthcare providers to respond affirmatively without stigmatization are urgently needed for MSM in these countries. Disclosure only to a family member was linked to perceived and enacted healthcare stigma, illustrating how disclosure to family is complexly linked to experiences of stigma in other contexts even when disclosure has not explicitly occurred within that context. Negative reactions to disclosures of same-sex practices from family may prime men to perceive potentially benign interactions in healthcare or other contexts negatively and ascribe those interactions to their sexuality, even if the men have not actually made such disclosures, particularly if men have high rejection sensitivities [54]. This could account for the link between disclosure and the nonspecific feeling of having felt mistreated in a health center. MSM who have disclosed to family only may simultaneously hold a less-concealable sexual or other identity or status, increasing their vulnerability to enacted stigma in other contexts, despite having not disclosed their same-sex practices in those contexts [23], or they may be marginalized in some other way that we did not measure that resulted in enacted healthcare stigma.

Similarly, disclosure only to a HCW was linked to perceived and enacted healthcare stigma. For MSM who have disclosed their same-sex practices to a HCW, the nonspecific feeling of having been mistreated could arise from a range of experiences, including overt enacted stigma, such as gossip or denial of services; more subtle forms of enacted stigma, such as microaggressions [55, 56]; or relatively benign interactions for MSM with high rejection sensitivities [54]. Sexuality-based stigma may also intersect with HIV stigma, increasing the likelihood of stigmatization [12, 57]. That disclosure only to a HCW was linked to enacted stigma reflects prior research that has consistently shown an association between disclosure and a range of enacted healthcare stigmas, including denial of services and verbal harassment, adding to the range of obstacles with which MSM must contend when seeking appropriate sexual health services [7, 9, 20–23].

Disclosure only to a HCW was also linked to having feared and avoided healthcare services (anticipated stigmas), but only in Cameroon. Same-sex practices remain highly stigmatized and criminalized in Cameroon, making disclosure of same-sex practices particularly risky there [58–62], though there have been increased efforts to address these issues in recent years [63]. MSM who have experienced or perceived negative reactions to their disclosure of same-sex practices or who are aware of the potential for these reactions may fear and avoid situations where such reactions are possible, including healthcare situations [54, 64], as has been demonstrated in other sub-Saharan African countries [13, 26, 31, 65, 66]. That Cameroon appeared to drive these associations could be indicative of a more unique context of stigmatization relative to the other countries, requiring a more tailored approach to stigma-mitigation. Multilevel stigma-mitigation interventions, such as a recently implemented three-tiered integrated stigma mitigation intervention in Senegal [67], could be particularly impactful in Cameroon.

We did not find a synergistic effect of disclosure to both a family member and HCW on experiences of healthcare stigma, possibly due to insufficient power to detect such an effect, with relatively small sample sizes in some countries, as well as small cell values for experiences of both disclosure and stigma from low endorsement in some cases. This lack of synergism could also indicate that family and healthcare stigmas operate independently, with family stigma not detracting from welcoming, affirming healthcare experiences. Alternatively, this lack of synergism could reflect a certain resilience and resistance to experiences of healthcare stigma. For MSM who have disclosed or are out about their same-sex practices in multiple contexts, sexuality may hold more salience and valence for their sense of self and identity, which may be protective against healthcare stigma [68]. Similarly, the overall low endorsement of healthcare stigmas – regardless of disclosure status – is significant. Being able to avoid stigmatizing experiences in healthcare settings, particularly in regions where same-sex practices are stigmatized and even criminalized, may reflect a certain degree of resilience among participants, undergirded by social cohesion and community support [46, 69, 70].

Findings should be considered in light of several limitations. We only assessed verbal disclosure of same-sex practices. Recent research has shown that behavioral disclosure by MSM, conveyed via gender nonconforming mannerisms, may incur more stigma than verbal disclosure of same-sex practices [27]. Future research that assesses both disclosures may be useful for better understanding how each is differentially or synergistically associated with stigma. An intersectional approach will be appropriate in such research, as other stigmas – such as those based on class and income, HIV status, or other attributes, which may require disclosures of their own – may intersect with sexuality- and gender-based stigmas. For example, MSM who engage in same-sex practices, display gender-nonconforming mannerisms, and are of low socioeconomic status may be stigmatized in ways that MSM of high socioeconomic status (but who are otherwise similar) are not, as the latter may have access to better sexual health services and providers, or may simply be treated better due to having higher income. Second, the directionality of the associations cannot be established due to the cross-sectional nature of the data. While it is perhaps more plausible that disclosure led to anticipating or experiencing stigma in healthcare, as was hypothesized and modeled in the present study, the opposite is also possible. For example, participants who disclosed their same-sex practices to a HCW and/or family may have feared or avoided healthcare prior to ever having made any disclosure about their sexuality simply because they have an awareness of the pervasive social stigma associated with same-sex practices in the context in which they live. Likewise, participants who were or felt mistreated by a HCW may have presumed it was due to assumptions about their undisclosed sexuality (or it may have been due to gender nonconforming behaviors) [27]; such a scenario may have prompted participants to confront the individual(s) they believed had stigmatized them, leading to their verbal disclosure of same-sex practices.

Additionally, social desirability bias may have influenced participant responses, as surveys were administered in-person in environments where same-sex practices are highly stigmatized. Fourth, the rarity of some country-specific disclosure patterns and experiences of healthcare stigma, combined with relatively small sample sizes, resulted in low precision of several parameter estimates and wide confidence intervals, potentially impacting the extent to which associations could be detected in some contexts. Lastly, respondent-driven and snowball sampling methods were used to recruit participants, limiting the generalizability of the findings. Moreover, the variation in disclosure and healthcare stigma experiences across countries observed in this study suggests there may be heterogeneous findings and unmeasured confounders (e.g., enforcement of laws targeting sexual minorities, social norms) from countries which were not studied, further complicating the extent to which generalizations can be made.

These findings underscore the need for not only the eradication of all forms of stigma and discrimination in healthcare contexts, but the presence of intentional affirmation of minority sexualities and marginalized persons. Supportive environments that enable safe disclosure are critical for providing appropriate sexual health services to MSM and maintaining their engagement in sexual health service utilization. For many MSM in countries across SSA, disclosure of same-sex practices lies at a critical intersection of tradeoffs, potentially leading to improved social support and sexual healthcare services (e.g., HIV pre-exposure prophylaxis access; improved counseling tailored to same-sex practices/partnerships) but also to stigmatization and victimization. Preventing sexuality-based stigma in healthcare settings requires fundamental change to heteronormative societal structures that perpetuate harmful norms and stereotypes, and maintain policies that stigmatize sexual minority men. Ultimately, advancing the HIV response in SSA and around the world necessitates multilevel interventions to mitigate community, institutional, and interpersonal-level stigma; foster enabling environments for disclosure of same-sex practices; and facilitate access to broader social and extrafamilial supports for MSM who have experienced stigma.

## Supplementary Information

**Additional file 1.**

## Data Availability

The datasets analyzed during the current study are not publicly available due to the sensitivity of the information collected but are available from the senior author (SDB) on reasonable request.

## Abbreviations

MSM: men who have sex with men
HIV: human immunodeficiency virus
SSA: sub-Saharan Africa
HCW: healthcare worker
RDS: respondent-driven sampling
