# Publisher Correction To: Disclosure of same-sex practices and experiences of healthcare stigma among cisgender men who have sex with men in five sub-Saharan African countries

**DOI:** 10.1186/s12889-021-12430-z

**Published:** 2022-01-12

**Authors:** John Mark Wiginton, Sarah M. Murray, Ohemaa Poku, Jura Augustinavicius, Kevon-Mark Phillip Jackman, Jeremy Kane, Serge C. Billong, Daouda Diouf, Ibrahima Ba, Tampose Mothopeng, Iliassou Mfochive Njindam, Gnilane Turpin, Ubald Tamoufe, Bhekie Sithole, Maria Zlotorzynska, Travis H. Sanchez, Stefan D. Baral

**Affiliations:** 1 Department of Health, Behavior & Society, Johns Hopkins University Bloomberg School of Public Health, 624 N Broadway Street, Baltimore, MD 21205 USA; 2 Department of Mental Health, Johns Hopkins University Bloomberg School of Public Health, Baltimore, MD USA; 3 Department of Epidemiology, Columbia University Mailman School of Public Health, New York City, NY USA; 4 Department of Public Health, Faculty of Medicine and Biomedical Sciences, University of Yaoundé I, Yaoundé, Cameroon; 5 Central Technical Group, National AIDS Control Committee, Yaoundé, Cameroon; 6 Enda Santé, Dakar, Senegal; 7 The People’s Matrix, Maseru, Lesotho; 8 Center for Public Health & Human Rights, Department of Epidemiology, Johns Hopkins University Bloomberg School of Public Health, Baltimore, MD USA; 9 Metabiota, Yaounde, Cameroon, Johns Hopkins Cameroon Program, Yaounde, Cameroon; 10 MbabaneFHI 360, Mbabane, Eswatini; 11 Department of Epidemiology, Emory University Rollins School of Public Health, Atlanta, GA USA

**Correction to: BMC Public Health 21, 2206 (2021)**

**https://doi.org/10.1186/s12889-021-12151-3**

During the publication of the original article [1], an error in the typesetting & publication process resulted in several wrong citations in the background, method & measures section.

The original article has been updated to rectify these errors.

The publisher apologizes for the inconvenience caused to the authors & readers.
