# Supplementary material for: Disclosure of same-sex practices and experiences of healthcare stigma among cisgender men who have sex with men in five sub-Saharan African countries
Source: BMC Public Health. 2021 Dec 3;21:2206. doi: 10.1186/s12889-021-12151-3 (PMC8641177; doi:10.1186/s12889-021-12151-3)
Supplement: Supplementary file 1 — Additional file 1. [file 12889_2021_12151_MOESM1_ESM.docx]

| Additional Table 1. Associations between disclosure of same-sex practices and healthcare stigma among MSM in SSA (pooled^†^), 2014-2016 (N=2,093). | | | | | | | | | | | | |
| --- | --- | --- | --- | --- | --- | --- | --- | --- | --- | --- | --- | --- |
|  | Afraid to seek health services | | | Avoided health services | | | Gossiped about by healthcare providers | | | Felt mistreated in a health center | | |
|  | Empty | OR  (95% CI) | aOR (95% CI) | Empty | OR  (95% CI) | aOR (95% CI) | Empty | OR  (95% CI) | aOR (95% CI) | Empty | OR  (95% CI) | aOR (95% CI) |
| Fixed effects | | | | | | | | | | | | |
| Same-sex practices disclosure | | | | | | | | | | | | |
| Not to a  FM or a  HCW (ref) | -- | 1.00 | 1.00 | -- | 1.00 | 1.00 | -- | 1.00 | 1.00 | -- | 1.00 | 1.00 |
| To a FM  only | -- | 1.07  (0.76, 1.52) | 1.10  (0.77, 1.57) | -- | 0.90  (0.62, 1.32) | 0.94  (0.63, 1.38) | -- | 1.47  (0.89, 2.41) | 1.57~  (0.94, 2.62) | -- | 1.28  (0.72, 2.31) | 1.52  (0.83, 2.79) |
| To a HCW  only | -- | 1.56*  (1.02, 2.39) | **1.62***  **(1.05, 2.50)** | -- | 1.67*  (1.08, 2.59) | **1.74***  **(1.11, 2.72)** | -- | 2.07*  (1.11, 3.89) | **2.01***  **(1.06, 3.81)** | -- | 3.77***  (1.97, 7.23) | **3.93*****  **(1.99, 7.74)** |
| To a FM  and a  HCW | -- | 2.25***  (1.45, 3.51) | **1.97****  **(1.24, 3.14)** | -- | 1.99**  (1.25, 3.17) | **1.76***  **(1.08, 2.87)** | -- | 3.85***  (2.23, 6.64) | **3.60*****  **(2.01, 6.44)** | -- | 2.85**  (1.48, 5.49) | **3.09****  **(1.53, 6.26)** |
| Random effect | | | | | | | | | | | | |
| Country-level variance (SE) | 0.43*** (0.32) | 0.41***  (0.30) | 0.34*** (0.25) | 0.53*** (0.39) | 0.51***  (0.37) | 0.42*** (0.31) | 0.36***  (0.28) | 0.36***  (0.28) | 0.26*** (0.21) | 0.39***  (0.30) | 0.43***  (0.35) | 0.26**  (0.23) |
| Model characteristics | | | | | | | | | | | | |
| Intra-class correlation | 11.6% | 11.2% | 9.4% | 13.9% | 13.3% | 11.4% | 10.0% | 9.8% | 7.3% | 10.5% | 11.7% | 7.3% |

^†^Cameroon, Lesotho, eSwatini, and Senegal; i.e., excluding Côte d’Ivoire

~p<0.10; *p<0.05; **p<0.01; ***p<0.001; significant, adjusted associations are also bolded

MSM, men who have sex with men; SSA, sub-Saharan Africa; OR, odds ratio; aOR, adjusted odds ratio; CI, confidence interval; FM, family member; HCW, healthcare worker

Controlling for age, education, income, HIV status, and depression

| Additional Table 2. Associations between disclosure of same-sex practices and healthcare stigma among MSM in SSA (pooled^†^), 2014-2016 (N=2,202). | | | | | | | | | | | | |
| --- | --- | --- | --- | --- | --- | --- | --- | --- | --- | --- | --- | --- |
|  | Afraid to seek health services | | | Avoided health services | | | Gossiped about by healthcare providers | | | Felt mistreated in a health center | | |
|  | Empty | OR  (95% CI) | aOR (95% CI) | Empty | OR  (95% CI) | aOR (95% CI) | Empty | OR  (95% CI) | aOR (95% CI) | Empty | OR  (95% CI) | aOR (95% CI) |
| Fixed effects | | | | | | | | | | | | |
| Same-sex practices disclosure | | | | | | | | | | | | |
| Not to a  FM or a  HCW (ref) | -- | 1.00 | 1.00 | -- | 1.00 | 1.00 | -- | 1.00 | 1.00 | -- | 1.00 | 1.00 |
| To a FM  only | -- | 0.93  (0.69, 1.23) | 0.94  (0.70, 1.26) | -- | 0.98  (0.71, 1.36) | 1.02  (0.73, 1.42) | -- | 1.60*  (1.07, 2.40) | **1.65***  **(1.09, 2.49)** | -- | 1.05  (0.58, 1.92) | 1.17  (0.63, 2.17) |
| To a HCW  only | -- | 1.41*  (1.00, 1.99) | 1.40~  (0.99, 2.00) | -- | 1.53*  (1.06, 2.22) | **1.56***  **(1.06, 2.28)** | -- | 1.23  (0.69, 2.16) | 1.17  (0.66, 2.09) | -- | 2.40**  (1.28, 4.50) | **2.30***  **(1.20, 4.41)** |
| To a FM  and a  HCW | -- | 1.50~  (0.99, 2.28) | 1.41  (0.92, 2.18) | -- | 1.52~  (0.97, 2.40) | 1.47  (0.91, 2.36) | -- | 4.09***  (2.52, 6.63) | **3.97*****  **(2.39, 6.61)** | -- | 3.13***  (1.67, 5.84) | **3.29*****  **(1.69, 6.42)** |
| Random effect | | | | | | | | | | | | |
| Country-level variance (SE) | 0.31*** (0.23) | 0.30***  (0.22) | 0.24*** (0.18) | 0.46*** (0.34) | 0.45***  (0.33) | 0.34*** (0.26) | 0.36***  (0.29) | 0.36***  (0.29) | 0.31*** (0.25) | 0.43***  (0.33) | 0.44***  (0.35) | 0.20**  (0.19) |
| Model characteristics | | | | | | | | | | | | |
| Intra-class correlation | 8.5% | 8.4% | 6.8% | 12.3% | 12.1% | 9.4% | 9.9% | 9.9% | 8.6% | 11.5% | 11.9% | 5.8% |

^†^Côte d’Ivoire, Lesotho, eSwatini, and Senegal; i.e., excluding Cameroon

~p<0.10; *p<0.05; **p<0.01; ***p<0.001; significant, adjusted associations are also bolded

MSM, men who have sex with men; SSA, sub-Saharan Africa; OR, odds ratio; aOR, adjusted odds ratio; CI, confidence interval; FM, family member; HCW, healthcare worker

Controlling for age, education, income, HIV status, and depression
